# Integration of Fuzzy AHP and Fuzzy TOPSIS Methods for Wire Electric Discharge Machining of Titanium (Ti6Al4V) Alloy Using RSM

**DOI:** 10.3390/ma14237408

**Published:** 2021-12-03

**Authors:** Kishan Fuse, Arrown Dalsaniya, Dhananj Modi, Jay Vora, Danil Yurievich Pimenov, Khaled Giasin, Parth Prajapati, Rakesh Chaudhari, Szymon Wojciechowski

**Affiliations:** 1 Department of Mechanical Engineering, School of Technology, Pandit Deendayal Energy University, Raysan, Gandhinagar 382007, India; kishan148fuse@gmail.com (K.F.); arrown.dind16@sot.pdpu.ac.in (A.D.); Dhananj.mind16@sot.pdpu.ac.in (D.M.); jay.vora@sot.pdpu.ac.in (J.V.); parth.prajapati@sot.pdpu.ac.in (P.P.); 2 Department of Automated Mechanical Engineering, South Ural State University, 454080 Chelyabinsk, Russia; danil_u@rambler.ru; 3 School of Mechanical and Design Engineering, University of Portsmouth, Portsmouth PO1 3DJ, UK; Khaled.giasin@port.ac.uk; 4 Faculty of Mechanical Engineering and Management, Poznan University of Technology, 60-965 Poznan, Poland

**Keywords:** wire electric discharge machining (WEDM), analytical hierarchy process (AHP), TOPSIS, response surface methodology (RSM), optimization

## Abstract

Titanium and its alloys exhibit numerous uses in aerospace, automobile, biomedical and marine industries because of their enhanced mechanical properties. However, the machinability of titanium alloys can be cumbersome due to their lower density, high hardness, low thermal conductivity, and low elastic modulus. The wire electrical discharge machining (WEDM) process is an effective choice for machining titanium and its alloys due to its unique machining characteristics. The present work proposes multi-objective optimization of WEDM on Ti6Al4V alloy using a fuzzy integrated multi-criteria decision-making (MCDM) approach. The use of MCDM has become an active area of research due to its proven ability to solve complex problems. The novelty of the present work is to use integrated fuzzy analytic hierarchy process (AHP) and fuzzy technique for order preference by similarity to ideal situation (TOPSIS) to optimize the WEDM process. The experiments were systematically conducted adapting the face-centered central composite design approach of response surface methodology. Three independent factors—pulse-on time (T_on_), pulse-off time (T_off_), and current—were chosen, each having three levels to monitor the process response in terms of cutting speed (V_C_), material removal rate (MRR), and surface roughness (SR). To assess the relevance and significance of the models, an analysis of variance was carried out. The optimal process parameters after integrating fuzzy AHP coupled with fuzzy TOPSIS approach found were T_on_ = 40 µs, T_off_ = 15 µs, and current = 2A.

## 1. Introduction

Titanium and its alloys possess a high strength-to-weight ratio which can be retained at high temperatures [1]. Owing to very high corrosion and erosion resistance, these metals are versatile in nature and they find applications in the pharmaceutical, aerospace, marine, chemical engineering, and food industries [2,3]. They have excellent bio-compatibility and as a consequence, they have been broadly utilized in biomedical applications and surgical implants [4]. Ti6Al4V alloy is used as an armor material for military vehicles, which require excellent ballistic performance. The titanium implants in a patient’s body permit examination with MRIs and NMRIs due to their non-ferromagnetic property [5,6]. However, titanium has very poor thermal conductivity, which leads to the localization of heat at the point of contact of the tool with the chip, resulting in high thermal gradients within the machining zone [7,8]. This in turn leads to increased tool wear rate and eventually tool failure. Moreover, titanium is chemically reactive at elevated temperatures, which may cause the tool to weld with the metal, leading to its premature failure [9]. Titanium has a low elastic modulus responsible for the deflection of job, chatter, and vibrations while machining. Given these limitations of conventional machining of titanium, there is a crucial need to explore the machinability of titanium with non-traditional processes, and wire electrical discharge machining (WEDM) is one of the process to investigate for effective machining.

The WEDM process is used to machine materials that are electrically conductive despite their mechanical properties [10,11,12]. The material removal in WEDM occurs with the input of the pulsating DC power supply, which discharges the electric current along the narrow inter-electrode gap between the workpiece and the tool [13,14,15]. The fine, clean, and precise cuts on the workpiece are possible because the wire, which acts as a cutting tool, possesses a small diameter and significant mechanical properties [16,17].

For sustainable manufacturing, the selection of optimum process parameters is indeed important. Several researchers worked on the optimization of the WEDM parameters for the machining of the Ti6Al4V alloy. The systematic design of experiments is essential to extract information from the experiments with minimal loss of resources in terms of time, money, raw materials, etc. Response surface methodology (RSM), Taguchi techniques using an orthogonal array, and fractional factorial are some of the methods for experimental design. The recorded output from the various experiments using one of the above methods is further used to optimize input process parameters. Multi-response optimization is needed in contemporary manufacturing. Various methods such as gray relational analysis (GRA) [11,15,18], heat transfer search (HTS) algorithm [2,19], teacher learning-based algorithm [20], particle swarm algorithm [21], genetic algorithm [22], artificial neural networks [23], etc., have been attempted to check their feasibility in finding the tread-off solution in terms of optimized process parameters.

Developing mathematical models of the WEDM process using various techniques is discussed in several studies. Mathematical models are helpful in predicting responses without performing extensive experiments. Sharma et al. [24] investigated WEDM parameters’ effect on dimensional deviation and cutting speed using a high-strength, low-alloy steel workpiece. They developed a mathematical relation of response surface using second-order CCD of RSM. Based on the mathematical model of cutting speed, they revealed that T_on_, T_off_, spark gap voltage, peak current, T_on_ × T_off_, T_on_ × IP, T_on_ × wire tension, and spark gap voltage × wire tension have significant effects on cutting speed. Furthermore, a mathematical model of dimensional deviation suggests that the main effects of T_on_, T_off_, SV, IP, and WT, and interaction effects between T_on_ and SV, T_on_ and IP are statistically significant for the analysis. Kavimani et al. [25] proposed a mathematical model for predicting the response data of MRR and surface roughness by using regression analysis with orthogonal array. The mathematical model proposed by Bose and Nandi [26] is based on two factors and four levels design of experiments on output response, such as surface roughness. They used desirable gray relational analysis algorithm for optimization of the WEDM process and model building. Daniel et al. [27] analyzed the effects of several WEDM process parameters on MRR and developed a mathematical model using regression analysis for predicting the response.

Arikatla et al. [28] optimized the parameters during the WEDM of titanium (Ti6Al4V) alloy using “RSM” method. The effects of pulse-on time (T_on_), wire tension, servo voltage, and pulse-off time (T_off_) were studied on the kerf width, surface roughness (SR), and MRR of the material. An analysis of variance (ANOVA) was conducted to check the impact of the process variables on the desired responses. Chaudhari et al. [29] attempted machining of Ni55.8Ti super-elastic shape memory alloy with the WEDM process. To exhibit the viability for industrial applications, a scientific approach consisting of RSM and HTS algorithm was planned and prepared for optimization. Saedon et al. [30] studied optimization of kerf and MRR during cutting of titanium (Ti6Al4V) alloy using WEDM. The experiments were performed with varying peak current, T_off_, wire tension, and wire feed. The design of the process parameters for the experiment was determined using Taguchi’s L9 design. The GRA approach was utilized for emphasizing the study for multiple performance characteristics. Payal et al. [31] employed the Taguchi fuzzy integration for parametric optimization of EDM process with multiple response measures. A fuzzy model was formed through which the optimal blend of parameters was obtained on the basis of multi-performance fuzzy index values.

Nowadays, multi-criteria decision making (MCDM) mechanisms are emerging in the manufacturing field. These are powerful tools for solving problems with multiple criteria. Some MCDM techniques help in assigning systematic weights to the criteria and some assign hierarchy of the alternatives [32]. Ananthakumar et al. [33] used the MCDM method, namely Technique for Order Preference by Similarity to Ideal Solution (TOPSIS), to determine the desired cutting conditions of plasma arc cutting. They defined the arc current of 45A, standoff distance of 2 mm, gas pressure of 3 bar, and cutting speed of 2400 mm/min as the best conditions for superior quality. Prabhu et al. [34] revealed that multiple quality characteristics of friction stir welded aluminum matrix composite can be improved using the TOPSIS approach. Tamjidy et al. [35] selected the best optimal solution in friction stir welding (FSW) of AA7075-T6 and AA6061-T6 using TOPSIS and Shannon entropy method. Sudhagar et al. [36] compared the ranking performance of TOPSIS and GRA for FSW of Al2024 aluminum alloy. The analytical hierarchy process (AHP) is an MCDM technique used to assign systematic weights to the criterion. Gaidhani and Kalamani [37] selected the most influential processing parameters in abrasive water jet machining based on weights obtained with the help of the AHP technique of MCDM. Babu and Venkataramaiah [38] optimized input parameters in WEDM of Al6061/SiCP composite using the AHP-TOPSIS approach. They performed experiments using Taguchi’s L18 design considering sensitivity, T_on_, wire feed rate (WF), T_off_, and wire type as input parameters. A similar study was performed using the AHP–TOPSIS hybrid approach during WEDM by Nayak and Mahapatra [39]. The current research on optimization is focused on developing a hybrid approach of optimization by integrating different MCDM techniques with a fuzzy approach. The fuzzy approach is a method of solving problems that are associated with uncertainty and vagueness. Different types of uncertainty can be observed in different optimization and decision-making problems. Chou et al. [40] used the fuzzy AHP and fuzzy TOPSIS to assess the performance of human resources in science and technology (HRST) in Southeast Asian countries. A fuzzy AHP approach was implemented to decide the preference weights for various performance measures while fuzzy TOPSIS was used to identify the best tread-off alternatives to accomplish the ideal HRST levels. They concluded that countries such as Singapore, South Korea, and Taiwan have better HRST performances compared to other Southeast Asian countries. Sirisawat and Kiatcharoenpol [41] employed the fuzzy AHP and fuzzy TOPSIS techniques to classify the reverse logistics barriers and also to prioritize and rank the solutions to implementing reverse logistics in the electronics industry. A hybrid decision-making method with a fuzzy approach was also found effective in deciding the best alternative in the manufacturing field. Roy and Dutta [42] studied the working of the EDM process considering duty cycle, current, T_on_, and gap voltage as controllable parameters. They used Taguchi’s L9 technique for experimental design and used integrated fuzzy AHP and fuzzy TOPSIS methods to determine the optimal set of controllable parameters. Furthermore, ANOVA analysis of closeness coefficient index (CC_i_) revealed that the current was the highest contributing factor among the selected parameters.

Previous studies suggest that many researchers attempted different MCDM techniques such as TOPSIS, AHP, fuzzy TOPSIS, and fuzzy AHP separately or in combination in different fields, including logistics, electronic industries, additive manufacturing, abrasive water jet machining, plasma arc cutting, etc. However, a study about investigating the use of response surface methodology using fuzzy AHP and fuzzy TOPSIS hybrid approach for WEDM machining of Ti6A4V has not yet been explored.

The present work intended to optimize the process parameters of T_on_, T_off_, and current during the WEDM of Ti6Al4V alloy. Cutting speed (V_C_), MRR, and SR were considered for the analysis as output response variables. The experiments were systematically designed using central composite design (CCD) of RSM and mathematical models were developed between responses and input parameters. The appropriateness of developed models was checked using ANOVA by analyzing the R-Seq values, lack-of-fit, and residual plots. Furthermore, the weights were assigned to considered criterion using fuzzy AHP, and optimal process parameter conditions were predicted using fuzzy TOPSIS. The confirmatory study was performed to verify the results of optimization.

## 2. Materials and Methods

### 2.1. Experimental Methodology

In the present study, a WEDM machine (Concord WEDM, DK7732, Concord Limited, Bangalore, India) was used to perform the experiments using Ti6Al4V as the work material (with a dimension of 90 × 50 × 6 mm^3^) and molybdenum wire (180 µm diameter) as the tool electrode. The dielectric fluid utilized was deionized water. Table 1 shows the chemical composition of the selected work material of Ti6Al4V. T_on_, T_off_, and current were selected as input process parameters based on recent literature to investigate their effects on WEDM machining in terms of cutting speed, MRR, and SR. Full factorial CCD of RSM was selected to design the experimental plan. CCD is very popular amongst multiple variants of RSM approaches because it offers great flexibility and allows sequential operations and effectiveness by providing the optimum solution in a minimum number of iterations. CCD design of RSM for three factors at three levels was implemented to prepare the experimental matrix. The levels of factors were selected based on preliminary trials and literature study. Table 2 shows the input parameters of the WEDM process at the three levels. Table 3 shows the full factorial CCD composed of the six axial and central points, and eight factorial points. During each experimental run, the wire was fed along a width direction of the work material. Each experiment was repeated three times; the average values of the three trials were reported and considered for the analysis. Minitab 17 software was utilized for the RSM design and analyzing the experimental data.

In total, 20 experiments were run randomly to avoid an experimental error. The output characteristic cutting speed refers to the speed in mm/min at which the wire cuts the workpiece. It is calculated using Equation (1):

(1) Vc=Lt

where *L* is the distance of 50 mm width of the workpiece which was cut in each pass and *t* is the machining time in minutes.

The material removal rate is considered as the volumetric material removed per unit time, which is calculated using Equation (2):

MRR = (wt._initial_ − wt._final_)/(ρ ∗ *t*) (2)

where wt._initial_ is the weight of the workpiece measured before the cut and wt._final_ is the weight of workpiece measured after the cut. ρ is the density of the titanium alloy, 4.42 gm/cm^3^. *t* is the cutting time measured for a particular cut in one minute.

Surface roughness is an important parameter that defines the quality of the machined surfaces. The SR of the WEDM part was determined with the use of the Surftest SJ-410 model. The arithmetic average roughness (Ra) value was determined in µm from cut specimens in the current study. The cutoff length of 0.8 mm and the evaluation length of 20 mm were used for the measurement of SR.

### 2.2. Fuzzy Analytical Hierarchy Process

**Figure 1:**
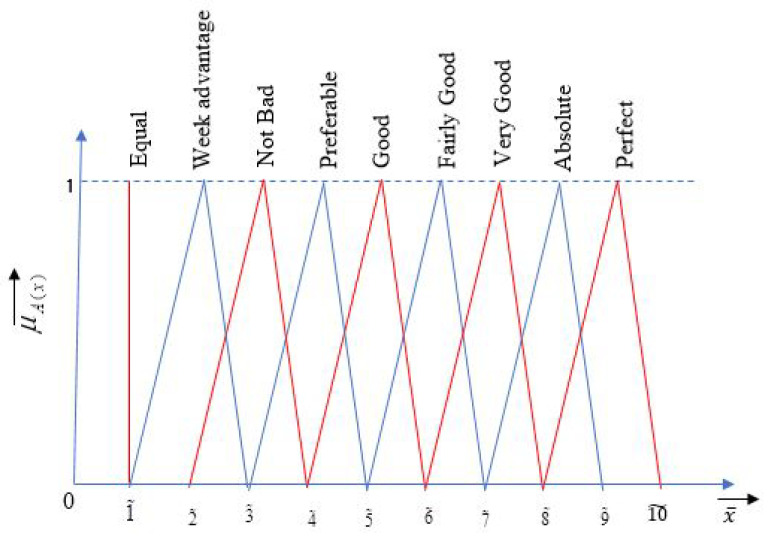
Membership function fuzzy scale for AHP along with linguistic variables [44].

**Figure 2:**
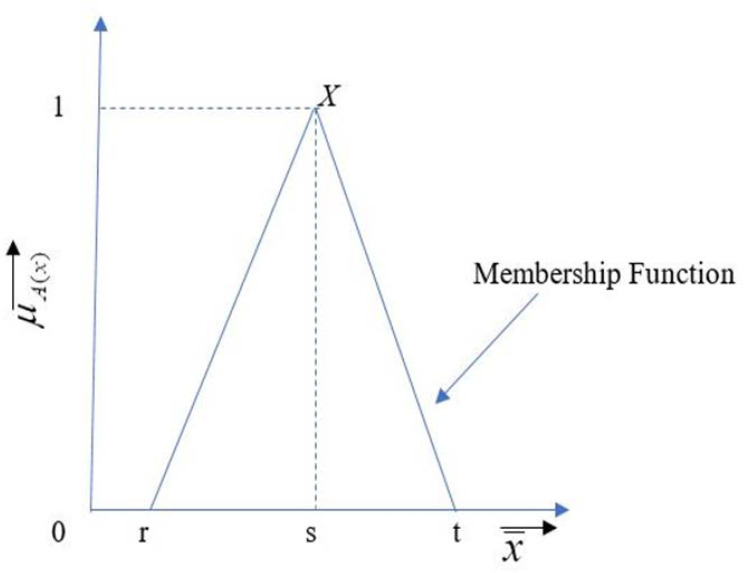
Triangular fuzzy number [45].

To use fuzzy TOPSIS for ranking of alternatives, each response needs to provide priority values or weights. These weights may vary from person to person. To overcome this limitation, T. L. Saaty [43] developed a technique called the analytic hierarchy process (AHP). This technique decomposes the decision-making situation into a systematic hierarchy of objectives, attributes, and alternatives. AHP is a compelling tool for complicated decision-making situations and helps decision makers define objectives and reach the best possible choice. By simplifying the complex choices to a progression of pairwise observations, and then integrating the outcomes, the AHP catches both abstract and target parts of a decision [44]. The limitation of conventional AHP is that it works with crisp information derived from linguistic responses. The scale of converting linguistic responses into crisp data in AHP is very unbalanced. The judgment of decision makers largely affects the result of conventional AHP due to aleatory uncertainty present in the natural language. This attracted researchers to integrate fuzzy theory with AHP and hence, fuzzy AHP was developed. The steps of fuzzy AHP in this research are as follows [42]:

Step 1: Construct the various leveled structure of objective, criterion, and alternatives of the problem.
Step 2: Construct a pairwise comparison matrix from the criteria/options available. Furthermore, assign linguistic terms using Figure 1 to the pairwise comparisons collected from decision makers. Convert linguistic terms into fuzzy numbers using Table 4. The generalized pairwise comparison matrix will be of the form shown in Equation (3).
  (3) $$\tilde{A}=\left(\begin{array}{ccc} 1 & \ldots & {\tilde{y}}_{1n}\\ \vdots & \ddots & \vdots \\ \frac{1}{{\tilde{y}}_{1n}} & \cdots & 1 \end{array}\right)$$
  where ${\tilde{y}}_{ij}$ = 1; *i* = *j*.
Step 3: Fuzzification is used to convert the linguistic term into a membership term. The fuzzification of the linguistic term can be possible using various functions such as triangular, bell-shaped, and trapezoidal functions. For this study, we used the triangular membership function, as shown in Figure 2. The assumed fuzzy numbers are shown in Equations (4) and (5).
  (4) X=(r,s,t)
  (5) Y=(l,m,n)
  where *r*, *s*, and *t* denote the lower, middle, and upper bounds of fuzzy number *X*, respectively, and *l*, *m*, and *n* denote the lower, middle, and the upper bounds of fuzzy number *Y*, respectively.
  Fuzzy weights can be found using fuzzy addition and multiplication [45]. The generalized fuzzy addition and fuzzy multiplication formulas are expressed by Equations (6) and (7).
  Fuzzy addition:
  (6) $$\tilde{X}⊕\tilde{Y}=(r,s,t)⊕(l,m,n)=(r+l,s+m,t+n)$$
  Fuzzy multiplication:
  (7) $$\tilde{X}⊗\tilde{Y}=(r,s,t)⊗(l,m,n)=(r\times l,s\times m,t\times n)$$
Step 4: Determine the fuzzy mean geometric value (FMGV) of each criteria using the geometric mean method. Equation (8) can be used for calculating FMGV. The fuzzy weights can be determined by using Equation (9).
  (8) $${\tilde{r}}_{j}={\left({\tilde{y}}_{j1}⊗{\tilde{y}}_{j2}⊗\ldots ⊗{\tilde{y}}_{jn}\right)}^{\frac{1}{n}}$$
  (9) $${\tilde{w}}_{j}={\tilde{r}}_{j}⊗{\left({\tilde{r}}_{1}⊕{\tilde{r}}_{2}⊕\ldots ⊕{\tilde{r}}_{n}\right)}^{-1}$$
  where ${\tilde{y}}_{ij}$ is the comparison of fuzzy value from criterion *i* to *j*; ${\tilde{r}}_{j}$ is the geometric mean value for comparison of the fuzzy value of criterion *j* to every other criterion; ${\tilde{w}}_{j}$ is the fuzzy weight of each criterion.

### 2.3. Fuzzy TOPSIS

Hwang and Yoon developed a multi-criteria decision analysis technique in 1981 called the Technique for Order of Preference by Similarity to Ideal Solution (TOPSIS). It was further modified by Yoon in 1987, and in 1993, it was further improved by Hwang Lai and Liu [46]. TOPSIS helps in choosing the best alternative with the least proximity from the positive ideal solution and the furthest from the negative ideal solution [47]. It is the strategy that compares the set of responses by identifying the contribution (weights) of each criterion. As the criteria are generally of random measurements, this might create problems in assessments. To avoid this situation, the need for fuzzy numbers is fundamental. Utilizing the fuzzy numbers in TOPSIS for multi-criteria decision making makes it easy for assessment [48]. The proposed steps for fuzzy TOPSIS implementation in this research are given below:

Step 1: Normalization of response: The normalization is important for converting measured outputs into the fuzzy number. The process of normalization was carried out considering the output based on the benefit criteria or the cost criteria. The V_C_ and MRR were normalized using the benefit criteria using Equation (10), whereas SR was normalized using the cost criteria using Equation (11).
  For benefit criteria:
  (10) $$r_{ij}\left(x\right)=\frac{x_{ij}-x_{min}}{x_{max}-x_{min}}$$
  For cost criteria:
  (11) $$r_{ij}\left(x\right)=\frac{x_{min}-x_{ij}}{x_{min}-x_{max}}$$
  where $r_{ij}\left(x\right)$ is the normalized value of output, $x_{max}$ is the maximum of $x_{ij}\;$ and $x_{min}$ is the minimum of $x_{ij}$.
  After normalization of the ratings, the responses of experiments were converted to fuzzy linguistic variables using Table 5. The five-level fuzzy linguistic variables are represented using a triangular fuzzy number [49].
Step 2: Fuzzification of normalized decision matrix: The decision matrix normalized in Step 1 can be converted to a fuzzified normalized decision matrix by assigning a sub-criteria grade to each alternative using Table 5 of the K membership function scale. Additionally, assign the weights to each sub-criteria grade.
  The weight of criteria:
  (12) $${\tilde{w}}_{j}^{k}=({\tilde{w}}_{j1}^{k},{\tilde{w}}_{j2}^{k},{\tilde{w}}_{j3}^{k})$$
Step 3: Calculate the weighted normalized fuzzy decision matrix: The weights obtained from fuzzy AHP are required to construct this matrix. The weighted normalized values can be calculated as:
  (13) $$\tilde{V}=({\tilde{v}}_{ij})\;\mathrm{Where}\;{\tilde{v}}_{ij}={\tilde{r}}_{ij}\times w_{j}$$
Step 4: Identify the positive ideal (V^+^) and negative ideal (V^−^) solutions: The fuzzy positive ideal solutions (FPIS, V^+^) and the fuzzy negative ideal solutions (FNIS, V^−^) must be calculated using Equations (14) and (15).
  $V^{+}=({\tilde{v}}_{1}^{+},{\tilde{v}}_{2}^{+},{\tilde{v}}_{3}^{+})$, where:
  (14) $${\tilde{v}}_{j}^{+}=\underset{i}{\max}\{v_{ij3}\}$$
  $V^{-}=({\tilde{v}}_{1}^{-},{\tilde{v}}_{2}^{-},{\tilde{v}}_{3}^{-})$, where:
  (15) $${\tilde{v}}_{j}^{-}=\underset{i}{\min}\{v_{ij1}\}$$
  Consideration of the maximum and minimum of V*_ij_* does not necessarily result in triangular fuzzy numbers, but we can obtain the ideal solutions as the fuzzy numbers using Equation (16).
  (16) $$d\left(\tilde{p},\;\tilde{q}\right)=\sqrt{\frac{1}{3}\left\{{\left(p_{1}-q_{1}\right)}^{2}+{\left(p_{2}-q_{2}\right)}^{2}+{\left(p_{3}-q_{3}\right)}^{2}\right\}}$$
  where $\tilde{p}$ = ($p_{1}$, $p_{2},\;p_{3}$) and $\tilde{q}$ = ($q_{1},\;q_{2},\;q_{3}$).
Step 5: Calculate separation measures: The separation measure $d_{i}^{+}$ is the summation of the distance of each response to the FPIS and $d_{i}^{-}$ is the summation of the distance of each response to the FNIS. The distance can be calculated by using the following equations.
  (17) $$d_{i}^{+}=\sum_{j=1}^{n}d({\tilde{v}}_{ij,}{\tilde{v}}_{j}^{+});\;i=1,\;2,\;\ldots ,\;m$$
  (18) $$d_{i}^{-}=\sum_{j=1}^{n}d({\tilde{v}}_{ij,}{\tilde{v}}_{j}^{-});\;i=1,\;2,\;\ldots ,\;m$$
Step 6: Calculate the similarities to the ideal solution: To solve the similarities, compute the closeness coefficient CC_i_ for each alternative [48].
  (19) $$CC_{i}=\frac{d_{i}^{-}}{d_{i}^{+}+d_{i}^{-}};\;CC_{i}\in [0,1]\;\forall i=1,\;2,\;\ldots ,\;n$$

## 3. Results and Discussions

### 3.1. Regression Equations

Evaluated values of the selected output response variables such as cutting speed, MRR, and SR are mentioned in Table 6. These measured response variables are then normalized using Equations (10) and (11) for further analysis. Table 6 shows the uncoded actual values of input process parameters as per “RSM” design. Minitab 17 software was used to find regression coefficients, and subsequently, ANOVA was assessed. The significance of the coefficients was tested at a 95% confidence level which is essential to recognize the most influencing model terms [50]. With the help of regression analysis, a mathematical relationship was obtained for V_C_, MRR, and SR in terms of input process variables. Equations (20)–(22) show the regression equations for V_C_, MRR, and SR, respectively.

The developed regression models consist of linear, quadratic, and interaction terms. It becomes is essential to study the significance of these terms on the output parameters. This can be executed by performing ANOVA. Thus, the ANOVA investigation was further studied to predict the significant terms of the proposed study. ANOVA tests the hypothesis based on the equality of means when several factors are considered. It is a statistical inference technique used to determine the influence of the hypothesis made for the model [51]. The study of the influence of input parameters at various levels is important for single-objective optimization. The main effect plots of the response highlight the optimum factor-level combinations for a given response. A detailed discussion is provided on mathematical model development, model adequacy checking, and the main effect plot in the following section for each response.

**V_C_** = 1.429 + 0.00567(T_on_) − 0.166(T_off_) + 1.613(Current) − 0.00005(T_on_ × T_on_) + 0.00233(T_off_ × T_off_) − 0.1699(Current × Current) + 0.000265(T_on_ × T_off_) + 0.001450(T_on_ × Current) − 0.01280(T_off_ × Current)                                            (20)

**MRR** = 1.37 + 0.0153(T_on_) − 0.118(T_off_) + 1.552(Current) − 0.000111(T_on_ × T_on_) + 0.00062(T_off_ × T_off_) − 0.1345(Current × Current) + 0.000267(T_on_ × T_off_) + 0.00183(T_on_ × Current) − 0.01450(T_off_ × Current)                                             (21)

**SR** = −1.48 + 0.1273(T_on_) − 0.341(T_off_) + 2.66(Current) − 0.000664(T_on_ × T_on_) + 0.0133(T_off_ × T_off_) − 0.318(Current × Current) − 0.00167(T_on_ × T_off_) + 0.01204(T_on_ × Current) − 0.0443(T_off_ × Current)                                           (22)

### 3.2. Analysis of Cutting Speed

**Figure 3:**
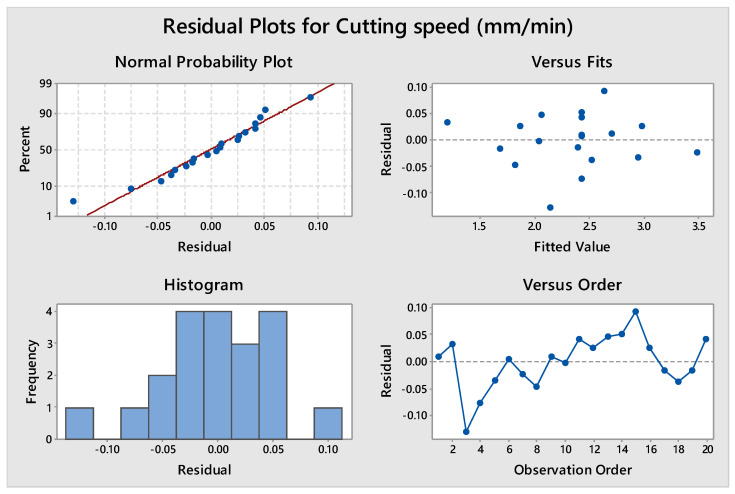
Residual plot for cutting speed.

ANOVA was carried out for cutting speed assuming a 95% confidence level. The ANOVA results are summarized in Table 7. F-value of the model (112.30) is much larger than the F-stat value (3.02). This suggests that the model is largely significant. There is just a 0.01% probability that a model F-value this large could be due to noise. The lack of fit F-value of 3.38 implies there is a 10.4% chance that a lack of fit F-value this large could be due to noise. For a confidence level of 95%, the *p*-value for any input parameter should be less than 0.05 to consider that parameter as significant [52,53]. It can be observed from Table 7 that all the input process variables have a *p*-value less than 0.05, which shows that all input process parameters are significant for V_C_. T_off_ shows the highest significance for obtaining higher value of V_C_ followed by current and T_on_. The *p*-value of the model is also less than 0.05, highlighting that the model is significant and best fitted for the selected range of process parameters. Insignificant lack-of-fit reveals the adequacy and fitness of the model [10]. The value of R^2^ indicates that 99.02% of the variation of cutting speed is contributed by the control factors and only 0.98% of total variation cannot be described by the quadratic model. The adjacent R-squared’ and predicted R-squared values of 98.14% and 94.04%, respectively, are in reasonable agreement. Figure 3 shows the normal probability plot of residuals for V_C_. ANOVA results are considered to be valid depending on the analysis of these plots. We observed that the developed regression model fit well with the observed values.

**Figure 4:**
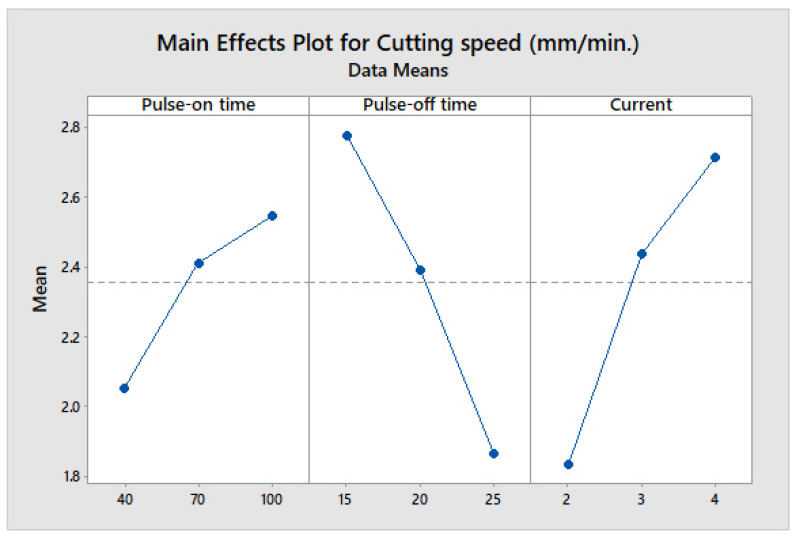
Main effect plot of cutting speed.

Figure 4 shows the influence of the three parameters at various levels on cutting speed. The increase in current elevates the discharge energy, which causes the rise in V_C_. The increase in the value of T_on_ signifies a rise in the duration of a spark, which causes the discharge energy to increase [2]. An increase in T_on_ and current significantly increases the spark intensity, which in turn escalates the melting and vaporization of the material from workpiece [7]. Hence, an increase in both T_on_ and current increases V_C_. However, a continuous decrease in the value of V_C_ has been observed with an increase in the value of T_off_ due to the absence of the spark during the machining [10].

### 3.3. Analysis of MRR

**Figure 5:**
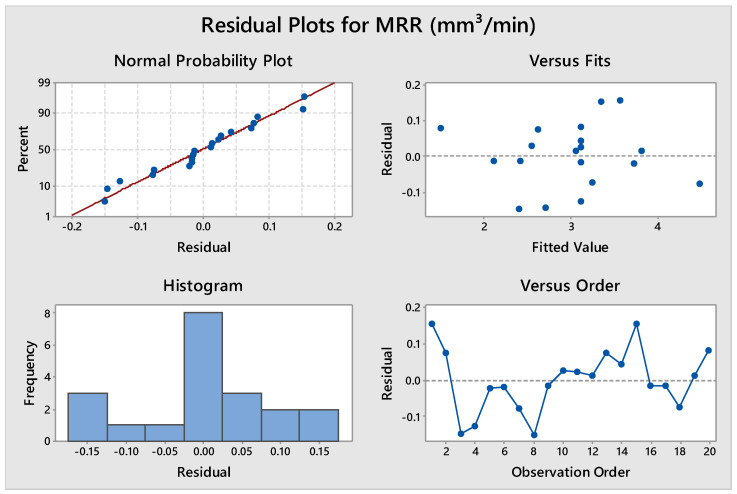
Residual plot for MRR.

The ANOVA results for the quadratic model developed between considered inputs and MRR at a 95% confidence level are shown in Table 8. The results indicate that the F-value of the model is 63.96 with a corresponding *p*-value of 0.000, which is less than 0.05, implying that the quadratic model is significant at the considered confidence level. A lack of fit of 4.46 with a corresponding *p*-value of 0.063 implies that it is not significant and suggests the fitness of the model. All the input process parameters (T_on_, T_off_ and current) show *p*-values of less than 0.05, suggesting that all are having a significant effect on MRR. The regression equation and ANOVA results of MRR revealed that MRR is significantly dependent on T_off_. T_off_ is most contributing factor (41.73%) on MRR followed by T_on_ and current. Saedon et al. [30] also reported T_off_ as the most significant factor (58%) while investigating WEDM machining on Ti-6Al-4V alloy. The R^2^ value of 98.29% indicates that 98.29% of the variation of MRR can be explained by the empirical model and only 1.71% of total variation cannot be described by the developed model. When predicted R^2^ and adjusted R^2^ values are in reasonable agreement, this confirms a strong correlation between observed and predicted values. Here, adjusted R^2^ is 96.76%, and the predicted value of R^2^ is 88.69%. The closeness of the values depicts a strong correlation between them. Figure 5 shows the residual plots for MRR in terms of normal probability plot versus residual, residual versus fitted values, histogram, and residual versus observation order. All the residual plots indicate that the regression model fits well with the observed values.

**Figure 6:**
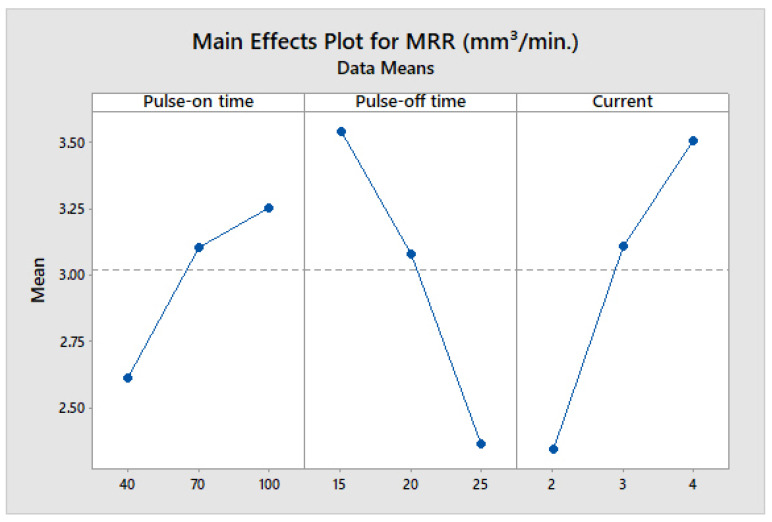
Main effect plot for MRR.

From Figure 6, it is inferred that with the rise in T_on_ and current, the influence of clearance form on MRR exhibits an increasing tendency. An increase in T_on_ and current significantly increases the spark intensity which in turn escalates the melting and vaporization of the material from the workpiece [10]. This further increases the MRR by a large amount. Saedon et al. [30] attributed increasing MRR with increasing T_on_ and current to the reduced dynamic shear strength of Ti alloy due to higher thermal influence in the machining region. The outcome of T_off_ on MRR shows a decreasing trend with a rise in T_off_ because of reduced spark ejection time and less MRR [9,54]. Thus, with an increase in T_off_, MRR is decreasing. Moreover, the slope indicates that it has a great effect on MRR; a slight increase in T_off_ leads to a decrease in MRR [9]. The positive dependency/correlation/relationship of MRR with T_on_ and negative dependency with T_off_ is also reported by Arikatla et al. [28].

### 3.4. Analysis of SR

**Figure 7:**
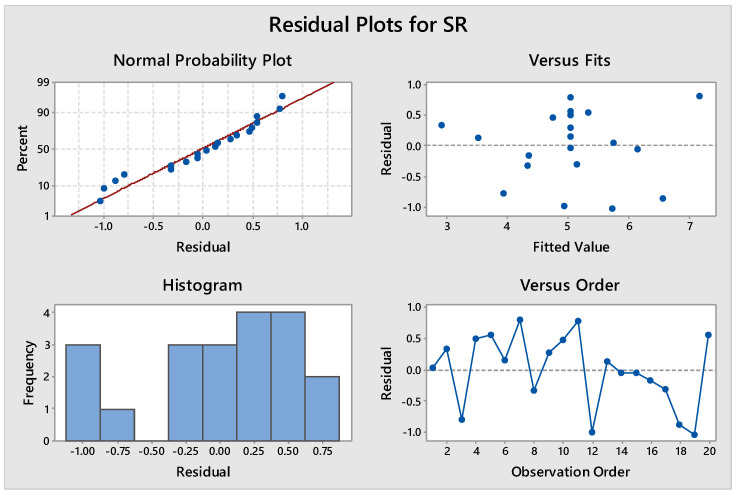
Residual plot for SR.

The ANOVA results for the quadratic model developed between considered inputs and machining time at a 95% confidence level are shown in Table 9. The results indicate that the F-value of the model is 11.67 with a corresponding *p*-value of 0.000, which is less than 0.05, implying that the quadratic model is significant at the considered confidence level. T_on_ and current were found to be the significant input process parameters for SR, with a higher contribution of T_on_ followed by current. The ANOVA results of SR revealed T_on_ as the main contributing factor (50.09%). Similarly, the largest contributing effect of T_on_ on SR was also reported by Arikatla et al. [28] A lack of fit of 3.73 with a corresponding *p*-value of 0.087 implies that it is not significant. The value of R^2^ of 0.9131 indicates that 91.31% of the variation of surface roughness can be explained by the empirical model and only 8.69% of total variation cannot be described by the developed model. When predicted R^2^ and adjusted R^2^ values are in reasonable agreement, it indicates strong relationship in observed and predicted values. Here, adjusted R^2^ is 0.9842 and the predicted value of R^2^ is 0.9473. Table 10 shows the model summary for all the responses. The closeness of the values depicts a strong correlation between them. Figure 7 shows the normal probability plot of residuals for SR. The plots highlight that that developed regression model fits well with the observed values.

**Figure 8:**
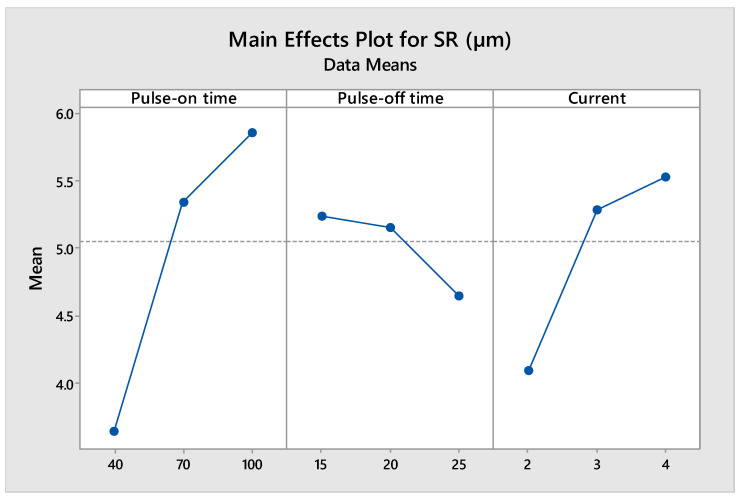
Main effect plot for SR.

Figure 8 shows the impact of machining parameters on SR. The effect of T_on_ is directly proportional to SR and has a constantly increasing trend because the higher the T_on_, the higher the energy of discharge and spark intensity, resulting in poor surface texture and vice versa [55]. This indicates that with the increase in T_on_, the SR value also increases. The effect of T_off_ on SR shows a reducing trend with growth in T_off_ (inversely proportional) [50,55]. Thus, with the increase in T_off_, SR is decreasing as discharge energy falls with the rise in T_off_, leading to the plunging of the crater dimensions and hence reducing SR. The effect of current on SR shows a growing tendency with a rise in current value and vice versa. Higher SR with the high current can be explained by the fact that crater sizes are also dependent on the ionization of the dielectric fluid and ionization takes place at faster rate with a higher current [50]. Thus, crater size increases with an increase in current value, resulting in higher SR. The positive dependency of SR on T_on_ and negative dependency on T_off_ was also reported by Arikatla et al. [28].

The ANOVA analysis of all three responses showed that developed mathematical models are significant for predicting the responses. It indicates that the experimental error is very minimal and collected output data can be used for multi-objective optimization.

The main effect plots of the considered responses (Figure 4, Figure 6 and Figure 8) can be used to identify optimum factor-level combinations from a single-objective optimization perspective. In the present study, V_C_ and MRR are of the “higher the better” category and SR falls under the “lower the better category”. Thus, the optimum parameter settings obtained considering a single objective are shown in Table 11. It can be observed that the optimum settings are conflicting in nature when all three responses are considered together. The industry demands process parameter setting, which can result in higher productivity (higher V_C_ and MRR) with good quality (low SR). This requires an improved means of optimization which can take care of such conflicting situations.

### 3.5. Optimization Using Integrated Fuzzy AHP and Fuzzy TOPSIS

#### 3.5.1. Fuzzy AHP

The hierarchical structure of the objective, criterion, and alternatives of the problem is constructed as per Step 1 of fuzzy AHP. The linguistic data of the pairwise comparison matrix were collected from domain experts and converted to crisp values using Table 4. The pairwise comparison matrix with crisp values was prepared using Equation (3) and is shown in Table 12.

The consistency ratio (CR) was calculated as 0.028. A value of CR less than 0.1 confirms good consistency in the judgments made by domain experts while assigning values in a pairwise comparison matrix [56]. Triangular fuzzy numbers from Table 4 were used for converting crisp values in the pairwise comparison matrix to fuzzy numbers. The obtained fuzzified comparison matrix is shown in Table 13.

The fuzzy mean geometric values were calculated using Equation (8) and were used to find fuzzy weights. The fuzzy weights of the criterion were calculated using Equation (9) and are shown in Table 14. These weights represent the lower, modal, and upper values of the fuzzy numbers, respectively.

#### 3.5.2. Fuzzy TOPSIS

In fuzzy TOPSIS, the alternative selection criteria were decided from the CCD design of the response surface methodology. Initially, the response data obtained were normalized using Equations (10) and (11) and are shown in Table 6. Then, a fuzzified normalized decision matrix was obtained by assigning weights to each sub-criteria grade using Equation (12). The fuzzified normalized decision matrix obtained is shown in Table 15.

The fuzzy weights obtained from the fuzzy AHP technique (Table 13) were then multiplied to each performance rating using Equation (13), resulting in a weighted normalized fuzzy decision matrix as shown in Table 16.

FPIS and FNIS were calculated using Equations (14) and (15). In the present study, V_C_ and MRR are benefit criteria and SR is cost criteria. Thus, we assumed FPIS $V_{j}^{+}$ as (1, 1, 1) for V_C_, MRR and (0, 0, 0) for SR. Additionally, we assigned FNIS $V_{j}^{-}$ as (0, 0, 0) for V_C_, MRR and (1, 1, 1) for SR.

Equations (17) and (18) were used to find FPIS and FNIS separation measures for each alternative, and the obtained results of separation measures are shown in Table 17. Finally, the closeness coefficient (CC_i_) was found for each alternative using Equation (19). The value of CC_i_ indicates whether the alternative is nearest to theoretical FPIS and furthest from the theoretical FNIS or vice versa [57]. The highest rank is given to the alternative with the highest value of the closeness coefficient, as shown in Table 17.

**Figure 9:**
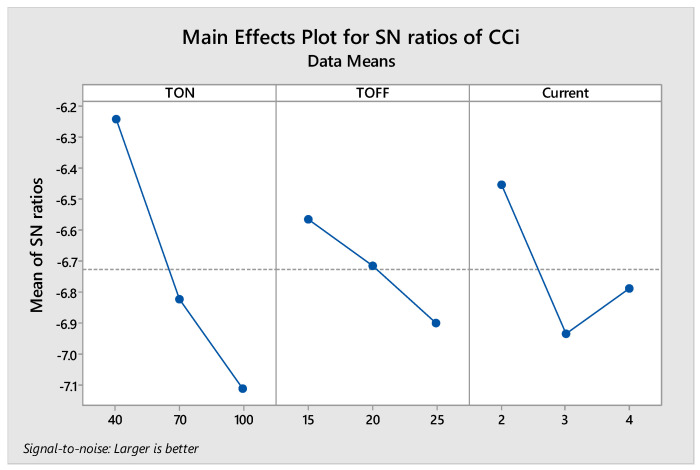
Main effect plot of S/N ratio of CC_i_.

After calculating the closeness coefficient using fuzzy TOPSIS, the optimized process parameters were determined using a single to noise (S/N) ratio of CC_i_ values. High CC_i_ values are always preferred. Hence, the (S/N) ratio was calculated using the “higher the better” strategy in Minitab. The obtained main effect plot of the (S/N) ratio is shown in Figure 9. The graph indicates the effect of factors’ concerning level. The optimum levels of process parameters were picked based on a higher value of the S/N ratio for the levels of factors. The optimized process parameters based on the highest values S/N ratio were a T_on_ of 40 µs, T_off_ of 15 µs, and current of 2A. The determined optimized process parameters correspond to alternative 12.

Confirmation experiments were performed to validate the optimal settings. Table 18 shows that the experimental results correlate well with the predicted results.

## 4. Conclusions

In this study, multi-criteria decision-making techniques, fuzzy AHP and fuzzy TOPSIS, were integrated for parameter optimization problems in Ti6Al4V alloy machining. The conclusions of the present work are summarized as follows:

- Response surface methodology is effective for systematically designing the experiments. The mathematical relations developed between dependent and independent parameters are significant for predicting the responses at a 95% confidence interval.
- ANOVA analysis confirmed that the input parameters T_on_, T_off_, and current significantly affect cutting speed, material removal rate, and surface roughness.
- Fuzzy AHP can be incorporated to prioritize the responses using data collected from experts. The use of the fuzzy approach eliminates the aleatory uncertainty present in the natural language. The weights calculated using fuzzy AHP can be incorporated in fuzzy TOPSIS without bias.
- For the considered range of process parameters, the optimal process parameters for WEDM are T_on_ = 40 µs, T_off_ = 15 µs, and current = 2A.
- The confirmatory experiments proved that fuzzy logic is an effective and efficient solution for the optimization of WEDM process parameters. The proposed integrated approach of RSM, fuzzy AHP, and fuzzy TOPSIS can be further extended for different machining processes.

## Figures and Tables

**Table 1:** Chemical composition (wt.%) of Ti6Al4V.

C	Fe	Al	N_2_	Cu	V	Ti
0.05	0.20	6.20	0.04	0.001	4.0	Balanced

**Table 2:** Input parameters with working range and their levels.

Parameter	Symbol	Unit	Level 1	Level 2	Level 3
Pulse-on time (T_on_)	A	µs	40	70	100
Pulse-off time (T_off_)	B	µs	15	20	25
Current	C	A	2	3	4

**Table 3:** Input parameters using a central composite design of RSM.

Std. Order	Run Order	T_on_	T_off_	Current
14	1	2	2	3
3	2	1	3	1
9	3	1	2	2
15	4	2	2	2
11	5	2	1	2
18	6	2	2	2
6	7	3	1	3
13	8	2	2	1
17	9	2	2	2
12	10	2	3	2
16	11	2	2	2
5	12	1	1	3
1	13	1	1	1
19	14	2	2	2
10	15	3	2	2
7	16	1	3	3
4	17	3	3	1
8	18	3	3	3
2	19	3	1	1
20	20	2	2	2

**Table 4:** Membership function [44].

Fuzzy Number	Linguistic Scale	Fuzzy Number		
9	Perfect	8	9	10
8	Absolute	7	8	9
7	Very good	6	7	8
6	Fairly good	5	6	7
5	Good	4	5	6
4	Preferable	3	4	5
3	Not bad	2	3	4
2	Weak advantage	1	2	3
1	Equal	1	1	1

**Table 5:** Transformation of fuzzy membership function [49].

Rank	Sub-Criteria Grade	Membership Function
Very Low (VL)	01	(0.00, 0.10, 0.25)
Low (L)	02	(0.15, 0.30, 0.45)
Medium (M)	03	(0.35, 0.50, 0.65)
High (H)	04	(0.55, 0.70, 0.85)
Very High (VH)	05	(0.75, 090, 1.00)

**Table 6:** Experimental results and normalized values of the output responses.

Run Order	T_on_ (µs)	T_off_ (µs)	Current (A)	Experimental Values			Normalized Values		
				V_C_ (mm/min)	MRR (mm^3^/min)	SR (µm)	V_C_	MRR	SR
1	70	20	4	2.715	3.730	5.80	0.6632	0.7597	0.5498
2	40	25	2	1.234	1.580	3.27	0.0000	0.0000	0.0249
3	40	20	3	2.012	2.560	3.15	0.3484	0.3463	0.0000
4	70	20	3	2.360	3.000	5.55	0.5043	0.5018	0.4979
5	70	15	3	2.917	3.710	5.89	0.7537	0.7527	0.5685
6	70	20	3	2.441	3.109	5.20	0.5405	0.5403	0.4253
7	100	15	4	3.467	4.410	7.97	1.0000	1.0000	1.0000
8	70	20	2	1.779	2.260	4.01	0.2441	0.2403	0.1784
9	70	20	3	2.444	3.110	5.33	0.5419	0.5406	0.4523
10	70	25	3	2.033	2.580	5.22	0.3578	0.3534	0.4295
11	70	20	3	2.477	3.150	5.83	0.5567	0.5548	0.5560
12	40	15	4	3.013	3.830	3.96	0.7967	0.7951	0.1680
13	40	15	2	2.114	2.690	2.98	0.3941	0.3922	0.1037
14	70	20	3	2.486	3.170	5.00	0.5607	0.5618	0.3838
15	100	20	3	2.731	3.500	6.10	0.6704	0.6784	0.6120
16	40	25	4	1.890	2.400	4.20	0.2938	0.2898	0.2178
17	100	25	2	1.673	2.100	4.83	0.1966	0.1837	0.3485
18	100	25	4	2.490	3.170	5.70	0.5625	0.5618	0.5290
19	100	15	2	2.381	3.080	4.71	0.5137	0.5300	0.3237
20	70	20	3	2.477	3.210	5.60	0.5567	0.5760	0.5083

**Table 7:** ANOVA for cutting speed.

Source	Sum of Squares	Df	Mean Sum of Square	F Value	*p*-Value	Contribution	Significance
Model	4.83875	9	0.53764	112.30	0.000	99.02%	significant
T_on_	0.61454	1	0.61454	128.37	0.000	12.58%	significant
T_off_	2.09032	1	2.09032	436.63	0.000	42.78%	significant
Current	1.93072	1	1.93072	403.29	0.000	39.51%	significant
T_on_ × T_off_	0.01264	1	0.01264	2.64	0.135	0.26%	
T_on_ × Current	0.01514	1	0.01514	3.16	0.106	0.31%	
T_off_ × Current	0.03277	1	0.03277	6.84	0.026	0.67%	significant
T_on_ × T_on_	0.00566	1	0.00566	1.18	0.302	0.12%	
T_off_ × T_off_	0.00929	1	0.00929	1.94	0.194	0.19%	
Current × Current	0.07935	1	0.07935	16.57	0.002	1.62%	significant
Residual	0.04787	10	0.00479			0.98%	
Lack of Fit	0.03694	5	0.00739	3.38	0.104	0.76%	Insignificant
Pure Error	0.01093	5	0.00219			0.22%	
Total	4.88662	19				100.00%	

**Table 8:** ANOVA for MRR.

Source	Sum of Squares	Degree of Freedom	Adjusted Mean Sum of Square	F Value	*p*-Value	Contribution	Significance
Model	8.17084	9	0.90787	63.96	0.000	98.29%	significant
T_on_	1.02400	1	1.02400	72.14	0.000	12.32%	significant
T_off_	3.46921	1	3.46921	244.39	0.000	41.73%	significant
Current	3.39889	1	3.39889	239.44	0.000	40.89%	significant
T_on_ × T_off_	0.01280	1	0.01280	0.90	0.365	0.15%	
T_on_ × Current	0.02420	1	0.02420	1.70	0.221	0.29%	
T_off_ × Current	0.04205	1	0.04205	2.96	0.116	0.51%	
T_on_ × T_on_	0.02723	1	0.02723	1.92	0.196	0.33%	
T_off_ × T_off_	0.00066	1	0.00066	0.05	0.834	0.01%	
Current × Current	0.04975	1	0.04975	3.50	0.091	0.60%	
Residual	0.14195	10	0.01420			1.71%	
Lack of Fit	0.11597	5	0.02319	4.46	0.0626	1.40%	Insignificant
Pure Error	0.02598	5	0.00520			0.31%	
Total	8.31279	19				100.00%	

**Table 9:** ANOVA for SR.

Source	Sum of Squares	Degree of Freedom	Mean Sum of Square	F Value	*p*-Value	Contribution	Significance
Model	22.3776	9	2.4864	11.67	0.000	91.31%	significant
T_on_	12.2766	1	12.2766	57.65	0.000	50.09%	significant
T_off_	0.8762	1	0.8762	4.11	0.070	3.58%	Not significant
Current	5.1266	1	5.1266	24.07	0.001	20.92%	significant
T_on_ × T_off_	0.5050	1	0.5050	2.37	0.155	2.06%	
T_on_ × Current	1.0440	1	1.0440	4.90	0.051	4.26%	
T_off_ × Current	0.3916	1	0.3916	1.84	0.205	1.60%	significant
T_on_ × T_on_	0.9825	1	0.9825	4.61	0.057	4.01%	
T_off_ × T_off_	0.3036	1	0.3036	1.43	0.260	1.24%	
Current × Current	0.2776	1	0.2776	1.30	0.280	1.13%	significant
Residual	2.1297	10	0.2130			8.69%	
Lack of Fit	1.6794	5	0.3359	3.73	0.087	6.85%	Insignificant
Pure Error	0.4503	5	0.0901			1.84%	
Total	24.5073	19				100.00%	

**Table 10:** Model summary for V_C_, MRR, and SR.

Response	Unit	Standard Deviation	R-sq	R-sq (adj)
V_C_	mm/min	0.0691912	99.02%	98.14%
MRR	mm^3^/min	0.119144	98.29%	96.76%
SR	µm	0.461485	91.31%	83.49%

**Table 11:** Optimum parameter setting considering single objective optimization.

Response	Unit	Optimum Parameter Setting Considering Single Objective Optimization
V_C_	mm/min	A_3_B_1_C_3_
MRR	mm^3^/min	A_3_B_1_C_3_
SR	µm	A_1_B_3_C_1_

**Table 12:** Comparison matrix.

	V_C_	MRR	SR
**V_C_**	1	1/3	1/7
**MRR**	3	1	1/4
**SR**	7	4	1

**Table 13:** Fuzzified comparison matrix.

	V_C_	MRR	SR
**V_C_**	(1, 1, 1)	(0.25, 0.33, 0.50)	(0.13, 0.14, 0.17)
**MRR**	(2, 3, 4)	(1, 1, 1)	(0.2, 0.25, 0.33)
**SR**	(6, 7, 8)	(3, 4, 5)	(1, 1, 1)

**Table 14:** Fuzzy weights.

	Weights
**V_C_**	(0.5286, 0.7049, 0.9312)
**MRR**	(0.1486, 0.2109, 0.2996)
**SR**	(0.0635, 0.0841, 0.1189)

**Table 15:** Fuzzified normalized data of the output responses.

Alternatives	V_C_ (mm/min)	MRR (mm^3^/min)	SR (µm)
1	(0.55, 0.70, 0.85)	(0.55, 0.70, 0.85)	(0.35, 0.50, 0.65)
2	(0.00, 0.10, 0.25)	(0.00, 0.10, 0.25)	(0.00, 0.10, 0.25)
3	(0.15, 0.30, 0.45)	(0.15, 0.30, 0.45)	(0.00, 0.10, 0.25)
4	(0.35, 0.50, 0.65)	(0.35, 0.50, 0.65)	(0.35, 0.50, 0.65)
5	(0.55, 0.70, 0.85)	(0.55, 0.70, 0.85)	(0.35, 0.50, 0.65)
6	(0.35, 0.50, 0.65)	(0.35, 0.50, 0.65)	(0.35, 0.50, 0.65)
7	(0.75, 0.90, 1.0)	(0.75, 0.90, 1.00)	(0.75, 0.90, 1.00)
8	(0.15, 0.30, 0.45)	(0.15, 0.30, 0.45)	(0.00, 0.10, 0.25)
9	(0.35, 0.50, 0.65)	(0.35, 0.50, 0.65)	(0.35, 0.50, 0.65)
10	(0.15, 0.30, 0.45)	(0.15, 0.30, 0.45)	(0.35, 0.50, 0.65)
11	(0.35, 0.50, 0.65)	(0.35, 0.50, 0.65)	(0.35, 0.50, 0.65)
12	(0.55, 0.70, 0.85)	(0.55, 0.70, 00.85)	(0.00, 0.10, 0.25)
13	(0.15, 0.30, 0.45)	(0.15, 0.30, 0.45)	(0.00, 0.10, 0.25)
14	(0.35, 0.50, 0.65)	(0.35, 0.50, 0.65)	(0.15, 0.30, 0.45)
15	(0.55, 0.70, 0.85)	(0.55, 0.70, 0.85)	(0.55, 0.70, 0.85)
16	(0.15, 0.30, 0.45)	(0.15, 0.30, 0.45)	(0.15, 0.30, 0.45)
17	(0.00, 0.10, 0.25)	(0.00, 0.10, 0.25)	(0.15, 0.30, 0.45)
18	(0.35, 0.50, 0.65)	(0.35, 0.50, 0.65)	(0.35, 0.50, 0.65)
19	(0.35, 0.50, 0.65)	(0.35, 0.50, 0.65)	(0.15, 0.30, 0.45)
20	(0.35, 0.50, 0.65)	(0.35, 0.50, 0.65)	(0.35, 0.50, 0.65)

**Table 16:** Weighted normalized data.

Alternatives	V_C_ (mm/min)	MRR (mm^3^/min)	SR (µm)
1	(0.034, 0.059, 0.101)	(0.082, 0.148, 0.255)	(0.185, 0.352, 0.605)
2	(0.000, 0.008, 0.030)	(0.000, 0.021, 0.075)	(0.000, 0.070, 0.233)
3	(0.010, 0.025, 0.054)	(0.022, 0.063, 0.135)	(0.000, 0.070, 0.233)
4	(0.022, 0.042, 0.077)	(0.052, 0.105, 0.195)	(0.185, 0.352, 0.605)
5	(0.035, 0.059, 0.101)	(0.082, 0.148, 0.255)	(0.185, 0.352, 0.605)
6	(0.022, 0.042, 0.077)	(0.052, 0.105, 0.195)	(0.185, 0.352, 0.605)
7	(0.048, 0.076, 0.119)	(0.111, 0.190, 0.300)	(0.396, 0.634, 0.931)
8	(0.010, 0.025, 0.054)	(0.022, 0.063, 0.135)	(0.000, 0.070, 0.233)
9	(0.022, 0.042, 0.077)	(0.052, 0.105, 0.195)	(0.185, 0.352, 0.605)
10	(0.010, 0.025, 0.054)	(0.022, 0.063, 0.135)	(0.185, 0.352, 0.605)
11	(0.022, 0.042, 0.077)	(0.052, 0.105, 0.195)	(0.185, 0.352, 0.605)
12	(0.035, 0.059, 0.101)	(0.082, 0.148, 0.255)	(0.000, 0.070, 0.233)
13	(0.010, 0.025, 0.054)	(0.022, 0.063, 0.135)	(0.000, 0.070, 0.233)
14	(0.022, 0.042, 0.077)	(0.052, 0.105, 0.195)	(0.079, 0.211, 0.419)
15	(0.035, 0.059, 0.101)	(0.022, 0.063, 0.134)	(0.291, 0.493, 0.792)
16	(0.010, 0.025, 0.054)	(0.022, 0.063, 0.135)	(0.079, 0.211, 0.419)
17	(0.000, 0.008, 0.030)	(0.000, 0.021, 0.075)	(0.079, 0.211, 0.419)
18	(0.022, 0.042, 0.077)	(0.052, 0.105, 0.195)	(0.185, 0.352, 0.605)
19	(0.022, 0.042, 0.077)	(0.052, 0.105, 0.195)	(0.079, 0.211, 0.419)
20	(0.022, 0.042, 0.077)	(0.052, 0.105, 0.195)	(0.185, 0.352, 0.605)

**Table 17:** Closeness coefficient index.

Alternatives	D+	D–	CC_i_
1	2.195	0.890	0.456
2	2.096	0.967	0.477
3	2.039	1.026	0.490
4	2.256	0.827	0.443
5	2.195	0.890	0.456
6	2.256	0.827	0.443
7	2.413	0.710	0.414
8	2.039	1.026	0.490
9	2.256	0.827	0.443
10	2.317	0.764	0.432
11	2.256	0.827	0.443
12	1.918	1.151	0.522
13	2.039	1.026	0.490
14	2.112	0.960	0.473
15	2.341	0.764	0.427
16	2.173	0.898	0.460
17	2.231	0.839	0.448
18	2.256	0.827	0.443
19	2.112	0.960	0.473
20	2.256	0.827	0.443

**Table 18:** Results of the confirmation experiment.

Performance Response	Optimal Setting	Predicted Values	Experimental Values	% Error
V_C_ (mm/min)	T_on_ 40 µs, T_off_ 15 µs, Current 2A	2.067	2.114	2.22
MRR (mm^3^/min)		2.616	2.690	2.75
SR (µm)		3.117	2.98	4.39

## Data Availability

Data presented in this study are available in this article.

## Nomenclature

AHP	Analytical hierarchy process
ANOVA	Analysis of variance
CC_i_	Closeness coefficient index
CCD	Central composite design
CR	Consistency ratio
FMGV	Fuzzy mean geometric value
FPIS	Fuzzy positive ideal solutions
FNIS	Fuzzy negative ideal solutions
GRA	Gray relational analysis
HTS	Heat transfer search
MCDM	Multi-criteria decision making
MRR	Material removal rate
RSM	Response surface methodology
S/N	Single to noise
SR	Surface roughness
TOPSIS	Technique for Order Preference by Similarity to Ideal Solution
T_on_	Pulse-on time
T_off_	Pulse-off time
V_C_	Cutting speed
WF	Wire feed rate
WEDM	Wire electrical discharge machining process
